# Cytotoxic activity of proteins isolated from extracts of *Corydalis cava *tubers in human cervical carcinoma HeLa cells

**DOI:** 10.1186/1472-6882-10-78

**Published:** 2010-12-17

**Authors:** Robert Nawrot, Maria Wolun-Cholewa, Wojciech Bialas, Danuta Wyrzykowska, Stanislaw Balcerkiewicz, Anna Gozdzicka-Jozefiak

**Affiliations:** 1 Department of Molecular Virology, Institute of Experimental Biology, Faculty of Biology, Adam Mickiewicz University, Umultowska 89, 61-614 Poznan, Poland; 2 Department of Cell Biology, University of Medical Sciences, Rokietnicka 5d, 60-806 Poznan, Poland; 3 Department of Biotechnology and Food Microbiology, Poznan University of Life Sciences, Wojska Polskiego 48, 60-627 Poznan, Poland; 4 Department of Plant Ecology and Environment Protection, Institute of Environmental Biology, Faculty of Biology, Adam Mickiewicz University, Umultowska 89, 61-614 Poznan, Poland

## Abstract

**Background:**

*Corydalis cava *Schweigg. & Koerte, the plant of numerous pharmacological activities, together with the studied earlier by our group *Chelidonium majus *L. (Greater Celandine), belong to the family *Papaveraceae*. The plant grows in Central and South Europe and produces the sizeable subterraneous tubers, empty inside, which are extremely resistant to various pathogen attacks. The *Corydalis sp. *tubers are a rich source of many biologically active substances, with the extensive use in European and Asian folk medicine. They have analgetic, sedating, narcotic, anti-inflammatory, anti-allergic and anti-tumour activities. On the other hand, there is no information about possible biological activities of proteins contained in *Corydalis cava *tubers.

**Methods:**

Nucleolytic proteins were isolated from the tubers of *C. cava *by separation on a heparin column and tested for DNase activity. Protein fractions showing nucleolytic activity were tested for cytotoxic activity in human cervical carcinoma HeLa cells. Cultures of HeLa cells were conducted in the presence of three protein concentrations: 42, 83 and 167 ng/ml during 48 h. Viability of cell cultures was appraised using XTT colorimetric test. Protein fractions were separated and protein bands were excised and sent for identification by mass spectrometry (LC-ESI-MS/MS).

**Results:**

The studied protein fractions showed an inhibiting effect on mitochondrial activity of HeLa cells, depending on the administered dose of proteins. The most pronounced effect was obtained with the highest concentration of the protein (167 ng/ml) - 43.45 ± 3% mitochondrial activity of HeLa cells were inhibited. Mass spectrometry results for the proteins of applied fractions showed that they contained plant defense- and pathogenesis-related (PR) proteins.

**Conclusions:**

The cytotoxic effect of studied proteins toward HeLa cell line cells has been evident and dependent on increasing dose of the protein. The present study, most probably, represents the first investigations on the effect of purified PR proteins from tuber extracts of a pharmacologically active plant on cell lines.

## Background

Plants from the family Papaveraceae are frequently used in traditional medicine as a remedy for treatment of several diseases. Such plants include *Corydalis cava *Schweigg. & Koerte; syn. *Corydalis bulbosa *(L.) Pers. non (L.) DC. and *Chelidonium majus *L. studied earlier by our group (Greater Celandine). The two species are closely related and belong to order Papaverales [1,2]. Milky sap as well as extracts of the whole *Chelidonium majus *plant has been used to treat papillae, warts, condylomae, which are a visible effect of human papilloma virus (HPV) infection. It has also been found that they have antimicrobial, antitumor, anti-inflammatory, antifungal, and fungistatic properties [3]. The tuber of *Corydalis sp. *contains isoquinoline alkaloids of apomorphine type, e.g. bulbocapnine, corydaline, which manifest analgetic, sedating and narcotic effects [4-7]. The plant has been used for the treatment of severe neurological disorders and mental diseases. It was also used in cases of asomnia, tension and anxiety conditions [7]. Some species of *Corydalis *are used in East Asia as analgetic drugs: in the traditional Chinese medicine, the species of *Corydalis yanhusho *was used to alleviate post-traumatic, colic, abdominal and menstrual pains [7]. Moreover, extracts of the same species showed anti-cancerous metastasis effect *in vitro *[8]. Anti-tumour activity of *Corydalis *species was also reported for Korean *Corydalis turtschaninovii*, which is effective for the treatment of inflammatory, allergic diseases and tumours [9]. Isoquinoline alkaloids contained in alcohol extracts of tubers in many species of *Corydalis*, affect metabolism of neurotransmitters [10]. Active compounds in such extracts include alkaloids, such as bulbocapnine, corydaline and corydine [11]. The similar curing properties of *Chelidonium majus *milky sap were attributed mainly to alkaloids, such as chelidonine, sanguinarine, berberine, coptosine, chelerythrine, and also several flavonoids and phenolic acids [12]. However, different findings show that all of them may be potentially toxic for human either alone or in combination [13,14].

Our earlier studies have shown that biological activity of *Chelidonium majus *milky sap may be related to its protein content. The majority of the identified proteins can be linked to direct and indirect stress and defense reactions, e.g. against different pathogens [15-17]. We have recently discovered that purified plant proteins from *Chelidonium majus *milky sap with nucleolytic activity, which probably belong to pathogenesis-related (PR) protein family, are capable of inducing apoptosis in human cervical cancer HeLa cells [17].

Therefore, the objective of our study was to evaluate the effect of purified proteins from extracts of *Corydalis cava *tubers with nucleolytic activity on HeLa tumour cells and to identify the protein content of such purified fractions using tandem mass spectrometry.

## Methods

### Plant material

*Corydalis cava *plants were collected during 2007 and 2008 in the neighbourhood of Poznań, Poland, during flowering, in April. A voucher specimen was deposited at the Department of Molecular Virology, Faculty of Biology, Adam Mickiewicz University in Poznan, Poland.

### Protein extract

The protein extract was prepared from tubers, dissolved in 0.1 M Tris-Cl buffer, pH 8.0, containing 10% glycerol (extract : buffer ratio was 1:1). The tuber extract (50% v/v) samples were separated into a supernatant, and a pellet fraction, by centrifugation at 12000 rpm for 20 min at 4°C as described in the protocol [16], with modifications. Supernatants were stored at -20°C for further analysis. Protein concentration was determined according to Lowry et al. [18].

### Isolation and purification of proteins

For isolation and purification of proteins, crude extracts from *C. cava *tubers collected in April were used. About 0.5-μg protein was loaded on HT Heparin column (GE Healthcare) (0.7 × 2.5 cm) equilibrated with 0.1 M Tris-HCl, pH 8.0, 10% glycerol. The column was eluted with a linear gradient of 0 to 2 M NaCl in the same buffer. The absorbance at 280 nm and DNase activity of all fractions (volume 1 ml) were determined.

### Analysis by SDS-PAGE

In order to verify the protein composition of chromatographic fractions, sodium dodecyl sulfate polyacrylamide gel electrophoresis (SDS-PAGE) was carried out in a slab mini-gel apparatus according to Laemmli 1970 [19], using 10% polyacrylamide as the separating gel and 5% polyacrylamide as the stacking gel. The proteins were reduced by heating them at 100°C in the presence of 2-mercaptoethanol for 5 min. After SDS-PAGE, the gels were fixed and stained with silver according to Shevchenko et al. [20].

### In-gel nuclease assay

An in-gel DNase assay was performed according to Thelen and Northcote [21], with modifications [22]. For in-gel DNase assay, *C. cava *tuber extracts or fractions after purification were dissolved in SDS-PAGE sample buffer without a reducing agent, incubated at 37°C for 10 min, and subjected to SDS-PAGE in 10% polyacrylamide gel containing denatured calf thymus DNA (40 μg/ml). After the electrophoresis and removal of SDS, the gels were washed with reaction buffer (10 mM Tris-HCl, pH 8.0, containing 10 mM CaCl_2_). DNase activity was visualized by staining the gel with ethidium bromide.

### Cell culture and viability assay

HeLa cells were cultured in RPMI 1640 supplemented with 5% FCS, 2 mmol/l L-glutamine, 100 μg/ml streptomycin and 100 U/ml penicillin. Viability of cell cultures was appraised using XTT colorimetric test, based on a dynamics of XTT (tetrazoline 2,3-bis(2-methoxy-4-nitro-5-sulfofenylo)-2H-5-carboxyanilide) stain reduction by viable cells with the formation of a colored product. Intensity of the fluorescence was measured at the wavelength of 450 nm. Percentage of mitochondrial metabolism activity inhibition was calculated according to the following equation: 100-(OD drug treated cells - OD medium alone/OD untreated cells-OD medium alone*100). Each experiment was conducted independently in 8 cultures and in each culture at least 200 cells were scored. Statistical analysis of the results involved the *t*-test for unpaired data using the STATISTICA ver.6.1 software. *P *value < 0.05 was considered to represent threshold of significance.

### LC-ESI-MS/MS analysis

Stained protein bands were excised from the gel and analyzed by liquid chromatography coupled to mass spectrometer in the Laboratory of Mass Spectrometry, Institute of Biochemistry and Biophysics, Polish Academy of Sciences, Warsaw, Poland. Samples were concentrated and desalted on RP-C18 precolumn (LC Packings, UK) and further peptide separation was achieved in a nano-HPLC RP-C18 column (LC Packings, 75 mM i.d.) of a UltiMate nano-HPLC system, using a 50-min linear acetonitrile gradient. Column outlet was directly coupled to Finningan Nanospray ion source of LTQ-FT (Thermo, USA) mass spectrometer working in the regime of data dependent MS to MS/MS switch. An electrospray voltage of 1500 V and a cone voltage of 30 V were used.

### MS/MS data analysis

The data were analysed automatically by database matching against the NCBInr protein database (NCBI, Bethesda, USA) with a Viridiplantae filter, using the MASCOT database search engine (Matrix science, London, UK; http://www.matrixscience.com) [23].

## Results

### Isolation and purification of proteins from *Corydalis cava *tuber extracts on a heparin column

**Figure 1:**
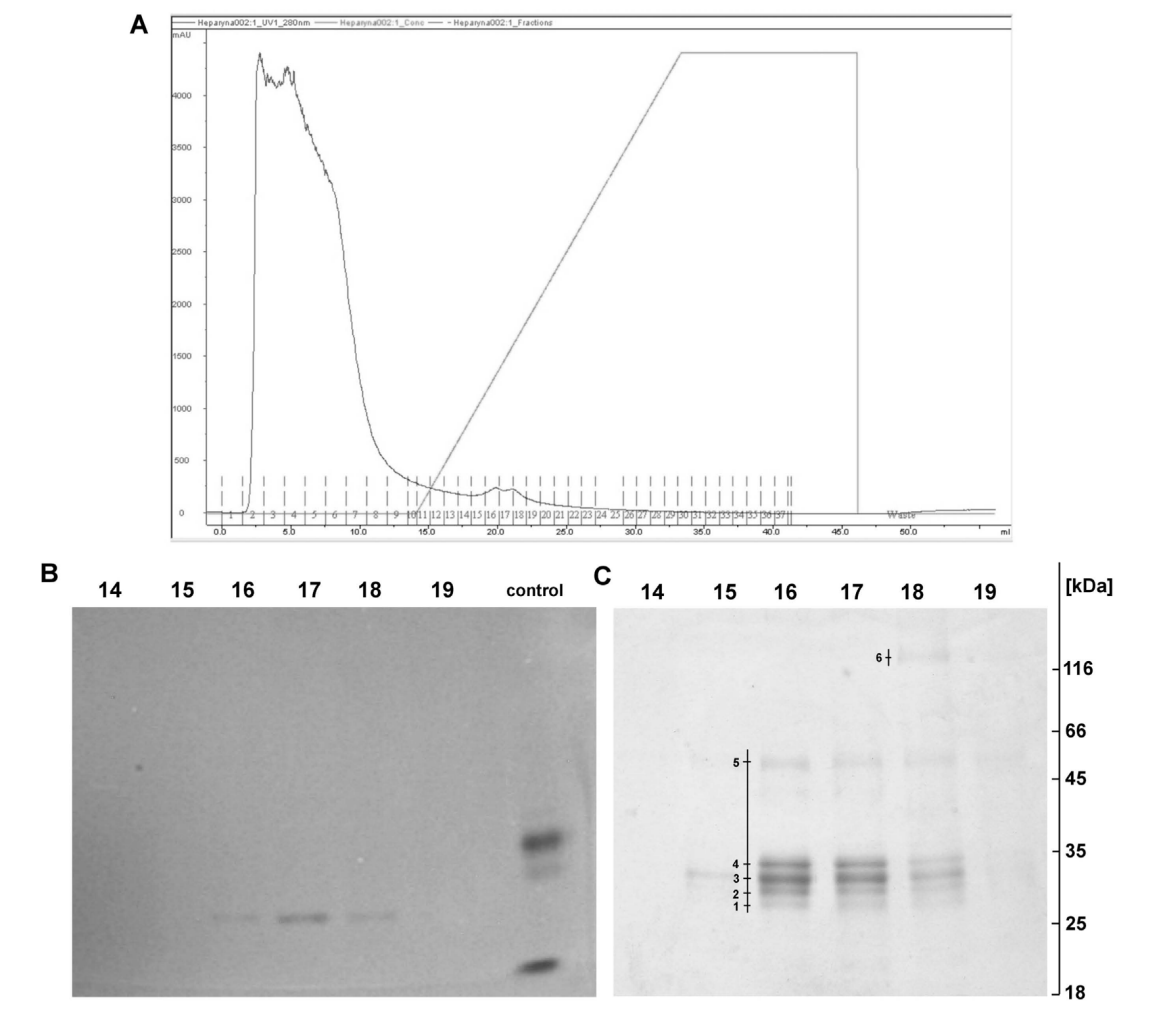
**Protein purification and electrophoretic analysis**. (A) Chromatographic profile of protein purification from *C. cava *tuber extracts in HT Heparin column (GE Healthcare). Fractions 1 to 9 represented flow-through fractions, 10 to 27 were eluted with a linear NaCl gradient (from 0 to 2 M). The absorbance of all fractions was measured at 280 nm and their DNase activity was estimated using in-gel assay. Proteins present in fractions 16, 17 and 18 were identified using LC-ESI-MS/MS. Fractions 16-19 following three rounds of purification were used in tests on HeLa tumour cell line. (B) In-gel DNase pattern of the gel in which DNase activity of protein fractions originating from *C. cava *tuber extracts was estimated. ssDNA containing 10% SDS-PAGE, following electrophoresis and incubation (12 h) in 10 mM Ca^2+^buffer, pH 8.0, was stained with ethidium bromide and viewed under UV light. Nucleolytic activity was noted in fractions Nos.16, 17 and 18 of MW around 30 kDa. Control: purified nucleases from *Chelidonium majus *milky sap served as positive control. (C) 10% SDS-PAGE following electrophoresis of fraction-contained proteins and silver staining according to Shevchenko et al. [20]. The separated fractions were identical to those in the gel in Fig. 1B. Fractions nos. 16 and 17 each contained 5 protein bands of MW around 30, 32, 35, 38 and 68 kDa, while fraction no. 18 contained an additional band of MW around 140 kDa. The protein bands were numbered 1-6, excised from the gel and sent for identification by mass spectrometry (LC-ESI-MS/MS). M - Protein Molecular Weight Marker (Fermentas).

In order to evaluate if biological activity of *Corydalis *tuber extracts is related to the proteins contained in them, the proteins of nucleolytic activity were isolated from the tubers by purification on a HT Heparin column (Figure 1A) using ÄKTA Explorer™ chromatographic system (Amersham Biosciences). Following the application of the extract to the column (0.7 × 2.5 cm), molecules which did not bind to the column were eluted using elution buffer (buffer A: 100 mM Tris, pH 8.0, 10% glycerol). The first eight fractions of 1.5 ml each represented the flow-through fractions. The subsequent fractions were eluted using a linear gradient of NaCl (from 0 to 2 M) (19 fractions of 1 ml each). In all the fractions, absorbance was measured at 280 nm (Figure 1A), indicating the presence of purified proteins in fractions 16-18. Protein fractions purified on heparin column were tested for DNase activity using in-gel DNase assay (Figure 1B) according to Thelen and Northcote [21]. Nucleolytic activity was demonstrated in fraction numbers 16, 17 and 18. All the fractions were also separated in SDS-containing polyacrylamide gel (SDS-PAGE), which was stained with silver according to Shevchenko et al. [20] (Figure 1C). Fraction numbers 16 and 17 each contained five protein bands of molecular weights (MW) approx. 30, 32, 35, 38 and 68 kDa, while the fraction number 18 contained an additional fraction of MW around 140 kDa.

### Analysis of mitochondrial activity of HeLa cells

All the fractions with nuclease activity were tested for cytotoxic activity in human cervical carcinoma HeLa cells. For the analysis, proteins contained in fraction numbers 16, 17, 18 and 19 were used, from three different rounds of purification. The buffer used for tuber extract isolation (0.1 M Tris, 10% glycerol), at the concentration of 26.7 μl/ml served as a negative control. For each fraction, 48 h cultures of HeLa cells were conducted in the presence of three protein concentrations: 42, 83 and 167 ng/ml from three purification rounds. HeLa cells on the plates were subjected to the action of XTT/PBS solution (1 mg XTT/ml in PBS + 1.53 mg PMS/ml in PBS) in order to estimate cell viability. Following incubation, the results were recorded using a small plate ELISA reader (Labsystems Multiskan MCC/340) with readout at 450 nm. The results are presented in Table 1.

**Table 1:** Mitochondrial activity inhibition in neoplastic HeLa cells under the effect of purified protein fractions from extracts of C. cava tubers.

Fraction no.	Protein concentration [ng/ml]	Mean value of HeLa cells inhibition [%]	Standard deviation [%]
16	42	4.55	3.53
16	83	15.57	2.42
16	167	35.31	4.32

17	42	8.66	5.19
17	83	22.35	2.40
17	167	39.15	3.34

18	42	12.59	5.37
18	83	32.42	3.13
18	167	43.45	3.06

19	42	5.90	5.43
19	83	15.18	7.10
19	167	21.78	6.06

Proteins from three rounds of purification were administered at concentrations of 42; 83; and 167 ng/ml. The data was analyzed using STATISTICA (version 6.1) software. [%] - inhibition of mitochondrial activity of HeLa cells under effect of examined proteins as compared to the control.

The cytotoxic effect of studied proteins in relation to HeLa cell line was clearly marked and it depended on the applied dose of protein: the most pronounced effect was obtained with protein fractions administered at the highest concentration (167 ng/ml), while at the intermediate concentration (83 ng/ml), the effect was appropriately lower compared to the highest concentration. Fraction 18 exerted the highest cytotoxic effect at the concentration of 167 ng/ml: 43.45 ± 3% cells underwent mitochondrial activity inhibition (Table 1). Protein fractions at the lowest protein concentration (42 ng/ml) manifested the least pronounced cytotoxic effect (e.g., fraction 16: 4.55 ± 3.5%, table 1). In the experiments, the control was provided by the pure elution buffer (0.1 M Tris, 10% glycerol), added to HeLa cells at the concentration of 26.7 μl/ml, which did not exert any effect on mitochondrial activity of HeLa cells.

### Identification of proteins in purified fractions by mass spectrometry

The protein bands were numbered 1 to 6 (Figure 1C), excised from the gel stained with silver [20] and sent for identification by mass spectrometry (LC-ESI-MS/MS) at the Laboratory of Mass Spectrometry, Institute of Biochemistry and Biophysics, Polish Academy of Sciences (IBB PAN) in Warsaw, Poland. The data was analyzed using MASCOT (http://www.matrixscience.com).

Results of protein identification using tandem mass spectrometry analysis (LC-ESI-MS/MS) are presented in Table 2. It contains a list of identified proteins from fractions of *Corydalis cava *tuber extracts. Results of the identification show that proteins contained in bands of fraction numbers 16-18 belong to plant pathogenesis- (PR) and defense-related proteins. Most of the identified proteins is directly or indirectly involved in defense reactions of the plant against various stresses, e.g. against attack of a pathogen (Table 2).

**Table 2:** Defense-related proteins identified in fractions after purification of nucleases from extracts of Corydalis cava tubers using LC-ESI-MS/MS.

No. of protein band^a)^	Identified protein^b)^	Accesion No.^c)^	Matched peptides^d)^	Score^e)^	Mol. mass (Da)^f)^	p*I*^g)^	Sequence coverage (%)^h)^
1, 2, 3, 4,	peroxidase [*Nicotiana tabacum*] (PR-9)	gi|14031049	16	560	39495	5.99	13

5, 6	heat shock protein 70 [*Chlorella zofingiensis*]	gi|18482472	4	180	70112	8.29	8

1	pectinesterase [*Phaseolus vulgaris*]	gi|21060	2	112	23967	9.52	4

2	SODP_PETHY Superoxide dismutase (ISS) [*Ostreococcus tauri*]	gi|116056311	2	74	42244	11.68	4

4	GRP-like protein 2 [*Gossypium hirsutum*]	gi|110559491	3	69	41333	6.01	8

5	disease resistance protein (TIR-NBS-LRR class), putative [*Arabidopsis thaliana*]	gi|15229962	1	64	117364	7.49	0

3	ribosomal protein S12 [*Mesostigma viride*]	gi|11466414	1	55	13815	11.32	9

1	DNA-binding protein [*Zea mays*]	gi|195658581	1	54	27597	6.49	4

4	chloroplast nucleoid DNA-binding protein-related [*Arabidopsis thaliana*]	gi|18391062	1	53	48429	7.48	2

4	RPP13-like protein [*Arabidopsis arenosa*]	gi|46410122	1	52	70976	5.51	2

a) Assigned numbers of protein bands as indicated in Fig. 1C.

b) Identified homologous proteins and organism from which it proceeds.

c) Database accession numbers according to: NCBInr (nr); trEMBL (trm).

d) Number of matched peptides with Mascot Search data (http://www.matrixscience.com).

e) Mascot Search Probability Based Mowse Score. Ions score is -10*Log(P), where P is the probability that the observed match is a random event. Individual ions scores > 48 indicate identity or extensive homology (p < 0.05).

f) Theoretical mass (kDa) of identified proteins. The values were retrieved from the protein database.

g) Theoretical pI of identified proteins. The values were retrieved from the protein database.

h) Amino acid sequence coverage for the identified proteins.

Analysis of DNase activity has demonstrated the presence of a band with nucleolytic activity in fractions 16, 17 and 18 of molecular weight around 30 kDa (Figure 1B). Comparison of SDS-PAGE gel following the separation of the same fractions and staining with the silver method (Figure 1C), demonstrates an analogous band of around 30 kDa molecular weight (No. 1) identified as DNA-binding protein from *Zea mays *with a very similar molecular weight of 27.6 kDa (Table 2). Thus, the studied fraction contains a number of proteins with defense and metabolic significance for the plant.

Table 2 lists identified defense-related proteins with plant species in which the proteins were detected as well as protein scores and sequence coverages. The number of compatible peptides for individual results ranged between 1 and 43, sequence coverage in a single case reached the maximum value of 26%. In the Table 2, numbers of protein bands were indicated in which particular proteins were identified (Figure 1C).

## Discussion

Plants that belong to family Papaveraceae, like Greater Celandine (*Chelidonium majus *L.) and *Corydalis cava *Schweigg. & Koerte, are a rich source of various biologically active substances with strong pharmacological activity. The mechanism of this activity is still unknown, but very important compounds are the proteins contained in the plants. Our earlier studies showed that biological activity of *Chelidonium majus *milky sap may be related to its protein content. The protein fractions which contained two nucleases induced apoptosis in human cervical cancer HeLa cells [17].

Most probably, the present study represents the first investigations on the effect of purified PR proteins from tuber extracts of a pharmacologically active plant on cell lines. The cytotoxic effect of studied proteins toward HeLa cell line cells has been evident and dependent on increasing dose of the protein.

Results of protein identification in fractions from purification of *C. cava *tuber extracts using LC-ESI-MS/MS analysis have shown that proteins contained in bands of fractions 16-18 belong to plant pathogenesis- and defense-related proteins, indirectly or directly involved in plant defense reactions against stresses of various kind. Many of them belong to the PR protein family involved in plant defense against the pathogen attack. The family includes 17 protein classes of variable activities [24]. *C. cava *tubers are exposed to the attack of fungal, viral, bacterial and other pathogens during the entire vegetation season as well as during winter time [25]. Accumulation of high number and variability of defense proteins in tubers together with several secondary metabolites, such as isoquinoline alkaloids, provides an effective and long-time protection against attack of pathogens. Moreover, defense-related proteins contained in *C. cava *tubers resemble 21 proteins identified earlier by our group in *Chelidonium majus *milky sap [15].

The studies represent preliminary demonstration of the effect exerted by tuber proteins from a plant of pharmacological significance on cells of a tumour cell line. The investigated protein fractions comprise a mixture of plant defense-related proteins. In subsequent studies, an effort will be made to isolate the individual proteins and examine their effect on tumour cells individually and in combinations as well. Nevertheless, the synergistic action of all the compounds present in plant protein extracts is very important for their activity. Many effective and extensively studied compounds of plant origin are, in fact, crude protein mixtures, such as phytotherapeutical drug bromelain, which is crude, aqueous extract from the stems and immature fruits of pineapples (*Ananas comosus *from the family Bromeliaceae), constituting a complex mixture of different thiol-endopeptidases and other components such as phosphatases, glucosidases, peroxidases, cellulases, glycoproteins, carbohydrates and several proteinase inhibitors [26]. Also, *Viscum album *L. extracts (VAE, European mistletoe) are composed of pharmacologically relevant compounds like: mistletoe lectins (ML I, II and III), viscotoxins and other low molecular proteins, VisalbCBA (*Viscum album *chitin-binding agglutinin), oligo- and polysaccharides, flavonoids, vesicles, triterpene acids, and others. Whole VAE as well as several of the compounds are cytotoxic and the mistletoe lectins have strong apoptosis-inducing effects [27].

Although the mechanism of presented activity of *C. cava *proteins is unknown, their defensive role for the plant could be very important. Many plant defense-related proteins belong to small (14-40 amino acids), linear, cationic peptides. These peptides have membrane lytic properties and potent activity against a broad spectrum of microorganisms. They organize into ordered secondary structures (α-helix and β-sheet) in the membrane [28]. Above a threshold concentration, peptides disturb the cell membrane and cause cell death due to membrane disintegration [29].

## Conclusions

Presented studies confirm that biological activity of *C. cava *extracts may also be related to proteins contained in the extracts. For possible further applications, the biologically active plant proteins should be separated from alkaloids and other secondary metabolites, which in higher doses might be toxic [14]. The studies represent preliminary demonstration of the effect exerted by tuber proteins from a plant of pharmacological significance on cells of a tumour cell line. In the subsequent studies, an effort will be made to isolate the individual proteins and examine their effect on tumour cells individually and in combinations.

## Competing interests

The authors declare that they have no competing interests.

## Authors' contributions

RN: Performed the study, collected plant material, evaluated the MS/MS data and prepared the manuscript; MWC: Carried out the study on cell lines, performed the statistical analysis and helped to draft the manuscript; WB: Isolated and purified proteins from plant extracts on a heparin column; DW: Participated in protein isolation and purification and in cell line analyses, helped in MS/MS data analysis; SB: Participated in design and coordination of the study, helped to collect the plant material; AGJ: Supervised the work, participated in its design and coordination and corrected the manuscript for publication. All the authors have approved the final manuscript.

## Pre-publication history

The pre-publication history for this paper can be accessed here:

http://www.biomedcentral.com/1472-6882/10/78/prepub

## References

[B1] Cronquist A An Integrated System of Classification of Flowering Plants 1981 New York, Columbia University Press

[B2] Reveal JL System of Classification. PBIO 250 Lecture Notes: Plant Taxonomy 1999 Department of Plant Biology, University of Maryland

[B3] Colombo ML Bosisio E Pharmacological activities of *Chelidonium majus *L. (*Papaveraceae*) Pharm Res 1996 33 127 134 10.1006/phrs.1996.0019 8870028

[B4] Rueffer M Bauer W Zenk MH The formation of corydaline and related alkaloids in *Corydalis cava in vivo *and *in vitro* Can J Chem 1994 72 170 175 10.1002/cjce.5450720128

[B5] Hänsel R Sticher O Pharmakognosie-Phytopharmazie 2004 Heidelberg, Springer Verlag

[B6] Freudenreich O Ocular side effects associated with dietary supplements and herbal medicines Drugs Today 2005 41 537 10.1358/dot.2005.41.8.896835 16234877

[B7] Cheng ZH Guo Y-L Wang H-Y Chen G-Q Qualitative and quantitative analysis of quaternary ammonium alkaloids from *Rhizoma Corydalis *by matrix-assisted laser desorption/ionization Fourier transform mass spectrometry coupled with a selective precipitation reaction using Reinecke salt Anal Chim Acta 2006 555 269 277 10.1016/j.aca.2005.09.003

[B8] Gao JL Shi JM He K Zhang QW Li SP Lee SM Wang YT Yanhusuo extract inhibits metastasis of breast cancer cells by modulating mitogen-activated protein kinase signaling pathways Oncol Rep 2008 20 819 824 18813823

[B9] An HJ Rim HK Chung HS Choi IY Kim NH Kim SJ Moon PD Myung NY Jeong HJ Jeong CH Chung SH Um JY Hong SH Kim HM Expression of inducible nitric oxide synthase by *Corydalis turtschaninovii *on interferon-gamma stimulated macrophages J Ethnopharmacol 2009 122 573 578 10.1016/j.jep.2008.12.030 19429329

[B10] Schäfer HL Schäfer H Schneider W Elstner EF Sedative action of extract combinations of *Eschscholtzia californica *and *Corydalis cava* Arzneimittelforschung 1995 45 124 126 7710431

[B11] Adsersen A Kjølbye A Dall O Jäger AK Acetylcholinesterase and butyrylcholinesterase inhibitory compounds from *Corydalis cava *Schweigg. & Kort J Ethnopharmacol 2007 113 179 182 10.1016/j.jep.2007.05.006 17574358

[B12] Tome F Colombo ML Distribution of alkaloids in *Chelidonium majus *and factors affecting their accumulation Phytochemistry 1995 40 37 39 10.1016/0031-9422(95)00055-C

[B13] Benninger J Schneider HT Schuppan D Kirchner T Hahn EG Acute hepatitis induced by greater celandine (*Chelidonium majus*) Gastroenterology 1999 117 1234 1237 10.1016/S0016-5085(99)70410-5 10535888

[B14] Stickel F Pöschl G Seitz HK Waldherr R Hahn EG Schuppan D Acute hepatitis induced by Greater Celandine (*Chelidonium majus*) Scand J Gastroenterol 2003 38 565 568 10.1080/00365520310000942 12795472

[B15] Nawrot R Kalinowski A Gozdzicka-Jozefiak A Proteomic analysis of *Chelidonium majus *milky sap using two-dimensional gel electrophoresis and tandem mass spectrometry Phytochemistry 2007 68 1612 1622 10.1016/j.phytochem.2007.03.039 17512564

[B16] Nawrot R Lesniewicz K Pienkowska J Gozdzicka-Jozefiak A A novel extracellular peroxidase and nucleases from a milky sap of *Chelidonium majus *L Fitoterapia 2007 78 496 501 10.1016/j.fitote.2007.04.012 17624685

[B17] Nawrot R Wołuń-Cholewa M Goździcka-Józefiak A Nucleases isolated from *Chelidonium majus *L. milky sap can induce apoptosis in human cervical carcinoma HeLa cells but not in Chinese Hamster Ovary CHO cells Folia Histochem Cytobiol 2008 46 79 83 10.2478/v10042-008-0011-x 18296268

[B18] Lowry OH Rosebrough NJ Farr AL Randall RJ Protein measurement with the Folin phenol reagent J Biol Chem 1951 193 265 275 14907713

[B19] Laemmli UK Cleavage of structural proteins during the assembly of the head of bacteriophage T4 Nature 1970 227 680 685 10.1038/227680a0 5432063

[B20] Shevchenko A Wilm M Vorm O Mann M Mass spectrometric sequencing of proteins silver-stained polyacrylamide gels Anal Chem 1996 68 850 858 10.1021/ac950914h 8779443

[B21] Thelen MP Northcote DH Identification and purification of a nuclease from *Zinnia elegans *L.: a potential molecular marker for xylogenesis Planta 1989 179 181 195 10.1007/BF00393688 24201517

[B22] Ito J Fukuda H ZEN1 is a key enzyme in the degradation of nuclear DNA during programmed cell death of tracheary elements Plant Cell 2002 14 3201 3211 10.1105/tpc.006411 12468737 PMC151212

[B23] Perkins DN Pappin DJ Creasy DM Cottrell JS Probability-based protein identification by searching sequence databases using mass spectrometry data Electrophoresis 1999 20 3551 3567 10.1002/(SICI)1522-2683(19991201)20:18<3551::AID-ELPS3551>3.0.CO;2-2 10612281

[B24] Sels J Mathys J De Coninck BM Cammue BP De Bolle MF Plant pathogenesis-related (PR) proteins: a focus on PR peptides Plant Physiol Biochem 2008 46 941 950 10.1016/j.plaphy.2008.06.011 18674922

[B25] Olesen JM Ehlers BK Age determination of individuals of *Corydalis *species and other perennial herbs Nord J Bot 2008 21 187 194 10.1111/j.1756-1051.2001.tb01356.x

[B26] Maurer HR Bromelain: biochemistry, pharmacology and medical use Cell Mol Life Sci 2001 58 1234 1245 10.1007/PL00000936 11577981 PMC11337410

[B27] Kienle GS Glockmann A Schink M Kiene H *Viscum album *L. extracts in breast and gynaecological cancers: a systematic review of clinical and preclinical research J Exp Clin Cancer Res 2009 28 79 10.1186/1756-9966-28-79 19519890 PMC2711058

[B28] Zhong J Chau Y Antitumor activity of a membrane lytic peptide cyclized with a linker sensitive to membrane type 1-matrix metalloproteinase Mol Cancer Ther 2008 7 2933 2940 10.1158/1535-7163.MCT-08-0528 18790774

[B29] Shai Y Mode of action of membrane active antimicrobial peptides Biopolymers 2002 66 236 248 10.1002/bip.10260 12491537

